# Selection on fish personality differs between a no‐take marine reserve and fished areas

**DOI:** 10.1111/eva.13242

**Published:** 2021-05-01

**Authors:** Susanna Huneide Thorbjørnsen, Even Moland, David Villegas‐Ríos, Katinka Bleeker, Halvor Knutsen, Esben Moland Olsen

**Affiliations:** ^1^ Centre for Coastal Research Department of Natural Sciences University of Agder Kristiansand Norway; ^2^ Institute of Marine Research, Flødevigen His Norway; ^3^ IMEDEA Instituto Mediterráneo de Estudios Avanzados (CSIC‐UIB) Department of Ecology and Marine Resources Ichthyology Group Esporles Balearic Islands Spain; ^4^ IIM Instituto de Investigaciones Marinas (CSIC) Department of Ecology and Marine Resources Fisheries Ecology Group Vigo Pontevedra Spain

**Keywords:** acoustic telemetry, harvest selection, home range, movement, personality, repeatability, salmonids, spatial ecology

## Abstract

Marine reserves can protect fish populations by increasing abundance and body size, but less is known about the effect of protection on fish behaviour. We looked for individual consistency in movement behaviours of sea trout in the marine habitat using acoustic telemetry to investigate whether they represent personality traits and if so, do they affect survival in relation to protection offered by a marine reserve. Home range size had a repeatability of 0.21, suggesting that it represents a personality trait, while mean swimming depth, activity and diurnal vertical migration were not repeatable movement behaviours. The effect of home range size on survival differed depending on the proportion of time fish spent in the reserve, where individuals spending more time in the reserve experienced a decrease in survival with larger home ranges while individuals spending little time in the reserve experienced an increase in survival with larger home ranges. We suggest that the diversity of fish home range sizes could be preserved by establishing networks of marine reserves encompassing different habitat types, ensuring both a heterogeneity in environmental conditions and fishing pressure.

## 1 INTRODUCTION

Fishing‐induced evolution and the consequences for populations have now been extensively documented (Kuparinen & Festa‐Bianchet, 2017). For example, selective fisheries may alter life‐history traits in a population by causing a shift towards maturation at earlier ages and smaller body sizes (Kuparinen et al., 2016; Olsen et al., 2004). However, fishing‐induced evolution of behaviour has received far less attention (Diaz Pauli & Sih, 2017). Interestingly, growth rate can be related to behavioural expression, and a selection regime targeting larger individuals may reduce the overall boldness in the population compared with a selection regime where small individuals are targeted (Biro & Post, 2008; Uusi‐Heikkilä et al., 2015). Harvesting may also select directly on behaviour (Uusi‐Heikkilä et al., 2008). For example, passive fishing gear can select against traits such as strong diel vertical migration (Olsen et al., 2012) and large home ranges (Alós et al., 2016) and lead to increased timidity (Arlinghaus et al., 2017), while active fishing gear such as trawling may favour bolder individuals (Andersen et al., 2018; Diaz Pauli et al., 2015). Moreover, since the vulnerability to certain harvest conditions may vary from one fish species to another (Killen et al., 2015), species‐specific information on behavioural responses to fishing restrictions and protection will be important for management.

Personality is consistent individual differences in behaviour over time and through contexts (Réale et al., 2007). Different behavioural strategies will be favoured in response to changes in a range of environmental variables, including food availability, population density and predator density, which is an important aspect in understanding their maintenance (Dingemanse & Réale, 2013). Assessing the heritability of behavioural traits can be difficult, and repeatability may be used as a proxy (Dochtermann et al., 2015). Previously, studies on repeatability of behaviour generally have been conducted in the laboratory, but more recently researchers have investigated repeatability of spatial behavioural traits also in the wild (Harrison et al., 2014; Villegas‐Ríos et al., 2017). Such studies are important for understanding how behavioural variation is maintained in nature, which in turn may provide useful input to adapting conservation strategies.

Marine reserves have long been used as a conservation tool to protect against depletion from fishing (Lester et al., 2009). However, it is unclear to what extent marine reserves may also help to preserve behavioural variation within populations by neutralizing fishing‐induced selection (Baskett & Barnett, 2015). Interestingly, marine reserves could drive unanticipated selection on behaviour due to their spatial configuration in relation to the spatial movements of the individuals, which might ultimately erode expected spillover benefits (Villegas‐Ríos et al., 2017). It is important thus to understand how selection may differ between harvested and protected areas and to what degree marine reserves may help in maintaining the behavioural diversity within populations, which ultimately represent resilience to environmental change (Dingemanse et al., 2004).

We used acoustic telemetry to quantify movement behaviour and its repeatability for anadromous brown trout (*Salmo trutta*) studied for up to 20 months in marine habitats in a Southern Norwegian fjord. We hypothesized that sea trout movement behaviour, here quantified as monthly averages of the movement metrics home range, mean swimming depth, activity and diurnal vertical migration, was repeatable among individuals and represented an aspect of their personality. Marine reserves may alter the fitness of the individuals depending on how and where they move. Therefore, we further hypothesized that selection on trout behaviour would differ between fished and protected areas.

## 2 MATERIALS AND METHODS

### 2.1 Study species

The brown trout (*Salmo trutta*) is a salmonid species in which anadromous populations are called sea trout. Sea trout have a highly variable life history with some individuals spending only the summer at sea, while others spend most of their time in marine areas only returning to the river to spawn during fall (Klemetsen et al., 2003). Marine migrations are motivated by access to more food, with important trade‐offs being adjustment to different salinities, increased energetic cost of movement and a potentially higher predation risk (Thorstad et al., 2016). The balance of these trade‐offs is likely an important part of the explanation for the diversity of migration strategies within populations (Thorstad et al., 2016) and population differentiation between streams (Knutsen et al., 2001; Olsen et al., 2006). In Norway, fishing for sea trout in marine habitats is recreational and permitted all year. Fishing can only be done using hook‐and‐line, except for one month in summer where specialized traps are permitted in the southern part of Norway. The minimum legal size for sea trout in the marine habitat in Norway is 35 cm. In the fjord, potential predators of sea trout are, among others, gulls, cormorants (*Phalacrocorax carbo*), harbour seals (*Phoca vitulina*) and gadids, as reported from a study system in western Norway (Jonsson & Jonsson, 2009).

### 2.2 Study system and data collection

**FIGURE 1:**
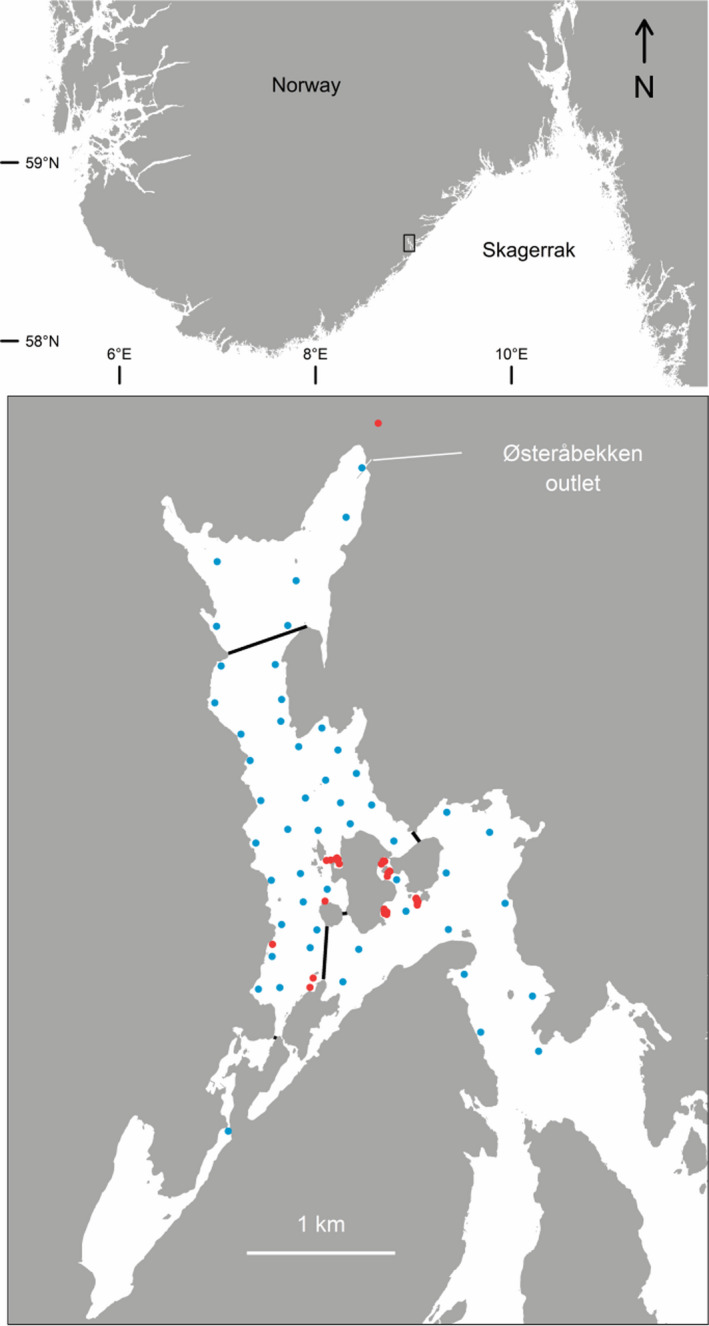
Map of the Tvedestrand fjord (below) and its location along the Norwegian Skagerrak coast (above). The marine reserve in the centre of the fjord is delineated with black lines. Blue dots represent receiver locations, and red dots represent capture locations

Movement data were collected in the Tvedestrand fjord (3.8 km^2^, max depth: 87 m) located in southern Norway between spring 2013 and fall 2017 (Figure 1). A telemetry array consisting of 50 Vemco VR2‐W receivers (VEMCO Ltd.) was deployed in the fjord, with the receivers being attached to moorings and kept at three metres depth aided by subsurface buoys (for more details, see Villegas‐Ríos, Réale et al., 2017). One receiver was located close to the spawning river, Østeråbekken, in order to monitor river migrations, and three receivers located in the outer part of the fjord served as a gate to detect individuals dispersing towards the outer fjord and sea areas. The high density of receivers ensured a good coverage of the fjord (see also Supporting Information, Thorbjørnsen et al., 2019). A marine protected area (1.5 km^2^) prohibiting all types of fishing was established within the spatial coverage of the telemetry array in 2012. Fishing is also prohibited in Østeråbekken and up to 100 m from the outlet of the stream.

Sea trout were caught around the centre islands of the fjord in 2013 (April: *n* = 3; May: *n* = 26; September: *n* = 24; November: *n* = 7), 2015 (June: *n* = 3; October: *n* = 14; November: *n* = 5) and 2016 (April: *n* = 4; May: *n* = 7) using a beach seine, and also by electrofishing in the spawning river at 11 November 2016 (*n* = 23). Beach seine was chosen in an attempt to minimize sampling‐induced selection of particular behavioural types (Olsen et al., 2012). Electrofishing was added as a complement to increase sample size in 2016. Individuals were anaesthetized with clove oil, and a transmitter was inserted in the abdominal cavity (for details, see Olsen et al., 2012). We used Vemco V9P and V13P transmitters, which had a maximum battery life of 508–696 and 1292 days, respectively. Signals were emitted with a random delay of 180 ± 70 s. Accuracy and resolution of depth measurements were ±2.5 m and 0.22 m, respectively, and max depth was 50 m or more for the different tags. Sea trout were not externally marked. Fin clips were taken for DNA analysis and preserved in 95% ethanol.

In total, 116 sea trout (mean body length: 337 mm, range: 215–635 mm) were caught, tagged and monitored in the Tvedestrand fjord during a 1669‐day study period (spring 2013–fall 2017). A total of 20 individuals were excluded from the study due to tag malfunction (*n* = 4), post‐surgical mortality (*n* = 5) or limited presence in the study area (<14 days, *n* = 11). Time spent in the study ranged from 1 to 20 months. Initial data exploration revealed that sex had no effect on any behavioural trait.

### 2.3 Data preparation and estimation of behavioural metrics

Detections were downloaded from the receivers and processed using the VUE software (VEMCO Ltd.), and further data preparation and analyses were done in the R environment (R Core Team, 2016). All detections after presumed death were censored, which was defined to have occurred when continuing detections indicated that horizontal and vertical movement had ceased (Olsen et al., 2012). Note that this could also represent transmitter loss. Fish were defined as dispersed after having followed a directional path out of the reserve with final detections occurring at the outermost receivers. Single detections within one day per fish were removed to eliminate potential code collisions and false detections, and above surface depth measurements were defined as NA. Four traits were used to describe the movement behaviour in the marine phase: home range, mean swimming depth, activity and diurnal vertical migration. Monthly replicates were used for all traits. Monthly 95% home ranges were calculated using locations based on position averages (PAVs, centres of activity), following Simpfendorfer et al. (2002). PAVs are weighted average locations within an array of receivers, based on the number of detections at each receiver during a specified time period (Simpfendorfer et al., 2002), in this case 30 min. Home ranges were then calculated from PAVs using kernel utilization distributions (bandwidth = 60, extent = 0.5) using the adehabitatHR package in R (Calenge, 2006). Depth measurements were averaged over months after removing replicated measurements occurring when a signal is detected at more than one receiver. Following Freitas et al. (2016), activity was defined as short‐term changes in depth and this was approximated as the standard deviation of depth per hour and then averaged over months. Diurnal vertical migration was calculated as the difference in mean depth from day to night within a calendar day and then averaged over months. Day and night phases were defined by solar elevation data obtained from the National Oceanic & Atmospheric Administration (NOAA) through the maptools package in R (Bivand & Lewin‐Koh, 2018). Only months where the fish was present in the fjord for a minimum of 15 days (not necessarily consecutive) were included in analyses.

### 2.4 Repeatability estimation

Univariate mixed‐effects models were fitted for each behavioural trait using the nlme package (Pinheiro et al., 2018) in R. For modelling purposes, home range and activity were log‐transformed to meet normality assumptions of the residuals. Monthly averages of each behavioural metric served as replicates for individual fish and individual sea trout identity was included as a random effect. We considered a trait to be repeatable when the inclusion of the random effect significantly improved the model fit. Provided that the random effect was supported, repeatability was calculated following Dingemanse and Dochtermann (2013) as:

(1) $$\text{Repeatability}=\frac{V_{{\text{ind}}_{0}}}{V_{{\text{ind}}_{0}}+V_{e_{0}}}$$

where $V_{{\text{ind}}_{0}}$ is the among‐individual variance and $V_{e_{0}}$ is the within‐individual variance. Model selection was done in two steps: (1) selecting the overall model structure by assessing if including the identity of the fish as a random effect and temporal autocorrelation between months improved the model (method = restricted maximum likelihood), followed by (2) selecting the fixed effects structure (method = maximum likelihood). Model selection was done using AIC‐values, and a minimum reduction of >2 units was required to assign significant improvement. When two or more models received similar support, the model with the simplest structure was selected. Fixed effects included in the models were body length (standardized to mean = 0, SD = 1), season (categorical variable with four levels, as defined by the UK calendar with spring starting on March 1), sex and capture location (two levels: fjord or river). Sex was determined using a sex‐determining marker loci based on Eisbrenner et al. (2014).

### 2.5 Survival

A survival curve was generated by computing a Kaplan–Meier estimator for right‐censored data (Cox & Oakes, 1984) using the ‘survival’ package in R (Therneau, 2015). Day of tagging was set to 0 for all individuals. Furthermore, a Cox proportional hazards regression model was used to assess the fixed effects of home range size and reserve use on survival. Reserve use was included in models either as the proportion of time spent in the reserve given that the fish was in the study area (calculated based on the location of PAVs), or as capture location (two levels: reserve or fished area), which served as a proxy for core area. Fish that were tagged on the river were excluded from this analysis. Both home range size and proportion of time spent in the reserve were calculated as the average of monthly estimates from tagging until death or the end of the study. Home range sizes were log‐transformed to meet normality assumptions. Additionally, the fixed effects fish length and season of capture (two levels: spring, as defined by the spring tagging season lasting from April to June, and fall, as defined by the fall tagging season lasting from September to November) were also included in all models. Model selection based on AIC was done in two steps: (1) selecting the best model structure related to the main variables of interest (home range size, proportion of time spent in the reserve, tagging location), including interaction effects between home range size and proportion of time spent in the reserve and home range size and capture location, and (2) selecting the best model related to the additional covariates body length and season of capture.

## 3 RESULTS

Home range size was a repeatable movement trait (repeatability = 0.21, Table S1), while mean swimming depth, activity and diurnal vertical migration were not (Tables S2–S4). Mean monthly home range size was 0.407 km^2^ (range: 0.065–2.14 km^2^), increased with body length, and was larger for fish caught in the fjord than fish caught in the river (Table 1, Figure S1). Home range size was also affected by season, being the largest in spring, followed by fall and summer, and the smallest in winter (Table 1).

**TABLE 1:** Summary of selected linear mixed‐effects and lme models explaining movement behaviour in sea trout

Response	Parameter	Estimate	SE	df	*p*‐value
Home range	Intercept	12.1	0.224	272	<0.001
	Length	0.134	0.0666	75	0.048
	Capture location, Fjord	0.604	0.224	75	0.0086
	Season, Winter	−0.342	0.123	272	0.0058
	Season, Spring	0.177	0.118	272	0.135
	Season, Summer	−0.145	0.111	272	0.193
	Variance, Intercept	0.3625			
	Variance, Residual	0.6945			
Mean depth	Intercept	0.678	0.331	324	0.0415
	Length	0.127	0.152	324	0.404
	Capture location, Fjord	0.996	0.326	324	0.0024
	Season, Winter	0.487	0.219	324	0.0268
	Season, Spring	0.881	0.203	324	<0.001
	Season, Summer	0.827	0.190	324	<0.001
	Season, Winter:Length	0.133	0.227	324	0.560
	Season, Spring:Length	0.441	0.213	324	0.0395
	Season, Summer:Length	0.671	0.206	324	0.0012
Activity	Intercept	−1.67	0.174	322	<0.001
	Length	0.251	0.0536	322	<0.001
	Capture location, Fjord	0.445	0.175	322	0.0114
	Season, Winter	−0.0953	0.105	322	0.364
	Season, Spring	0.317	0.0977	322	0.0013
	Season, Summer	0.339	0.0896	322	<0.001
Diurnal vertical migration	Intercept	0.00313	0.231	279	0.989
	Length	0.155	0.103	279	0.133
	Capture location, Fjord	0.475	0.224	279	0.0348
	Season, Winter	0.00867	0.156	279	0.956
	Season, Spring	0.716	0.144	279	<0.001
	Season, Summer	0.643	0.138	279	<0.001
	Season, Winter:Length	0.0835	0.155	279	0.590
	Season, Spring:Length	0.476	0.143	279	0.001
	Season, Summer:Length	0.280	0.140	279	0.046

Note

Associated parameter estimates, standard errors (SE), degrees of freedom (df) and *p*‐values are given.

Analysis of monthly mean swimming depth (mean = 2.27 m, range: 0.35–9.44 m) showed that fish caught in the fjord swam deeper than fish caught in the river (Table 1, Figure S1). Mean swimming depth was also affected by an interaction between fish body length and season. Mean swimming depth increased with body length and differed between seasons, with fish being located at more shallow depths during fall compared with all other seasons. The interaction between body length and season indicated a stronger positive effect of body length on mean swimming depth in summer, followed by spring, winter and fall (Table 1).

Activity (mean = 0.47 m, range: 0.018–3.67) increased with body length and was higher for fish caught in the fjord (Table 1, Figure S1). Activity differed between the seasons, and fish were most active during spring and summer, and least active during fall and winter (Table 1).

Diurnal vertical migration, the difference in mean depth from day to night (mean = 0.95 m, range: −0.75 to 5.08), was larger for fish caught in the fjord than fish caught in the river (Figure S1) and was affected by an interaction between body length and season (Table 1). Diurnal vertical migration increased with body length and differed between seasons, with fish having a larger daily movement span during spring and summer than in winter and fall. The interaction between body length and season indicated a stronger positive effect of body length on diurnal vertical migration in spring and summer than in winter and fall (Table 1).

Including autocorrelation led to significant improvement of all models with a behavioural trait as the response variable (Tables S1–S4).

**FIGURE 2:**
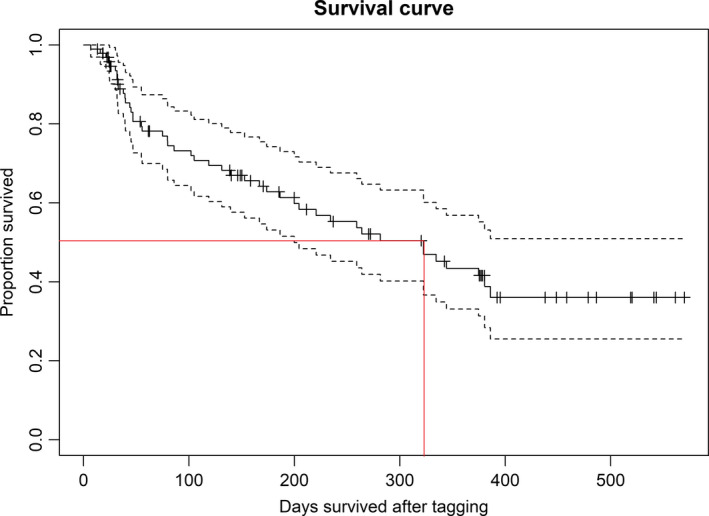
Right‐censored Kaplan–Meyer survival curve for sea trout in the Tvedestrand fjord. Red lines show median survival at 323 days. Tagging day was set to zero for all individuals. Vertical tick marks indicate right‐censored events where an individual was no longer tracked due to dispersal or end of study or battery life

**FIGURE 3:**
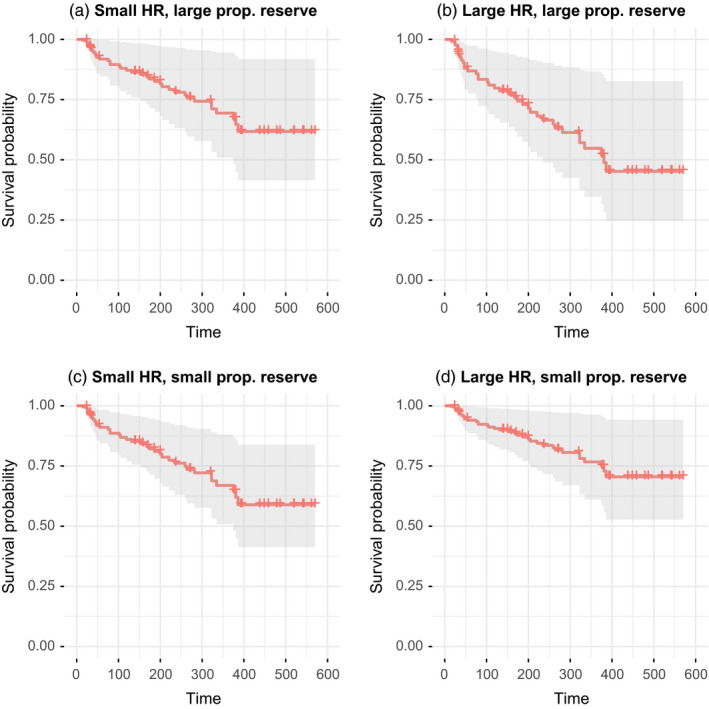
Survival curves for sea trout resulting from the Cox proportional hazards regression model with home range size, proportion of time spent in the reserve and season of tagging as explanatory variables. The four panels show different combinations of home range size and proportion of time spent in the reserve: (a) home range size = 0.265 km^2^, proportion of time in the reserve = 0.75; (b) home range size = 0.587 km^2^, proportion of time in the reserve = 0.75; (c) home range size = 0.265 km^2^, proportion of time in the reserve = 0.25; (d) home range size = 0. 587 km^2^, proportion of time in the reserve = 0.25. A home range size of 0.265 km^2^ corresponds to the 1st quartile of home range sizes, while a home range size of 0.587 km^2^ corresponds to the 3rd quartile of home range sizes. Season of tagging had value ‘fall’ in all survival curves

Estimated median survival was 323 days (10.8 months, Figure 2). At this point in the curve, estimated survival was 0.487 (95% CI 0.384–0.617). The best model predicting survival included average monthly home range size, proportion of time spent in the reserve and the interaction between these, in addition to season of tagging (Table S5). The effect of home range size on survival differed depending on the proportion of time spent in the reserve (Figure 3, Table 2). Larger home range sizes increased survival for individuals spending little time in the reserve whereas it decreased survival for individuals spending a large amount of time in the reserve. The effect of home range size on survival went from positive to negative when individuals spent more than 48% of their time inside the reserve. For example, model predictions showed that if an individual spent 25% of its time in the reserve, an increase in home range size from 0.265 km^2^ to 0.587 km^2^ would increase survival at day 386 (last recorded mortality event) by 20% (Figure 3). In contrast, model predictions showed that if an individual spent 75% of its time in the reserve, an increase in home range size from 0.265 km^2^ to 0.587 km^2^ would decrease survival at day 386 by 27%. A home range size of 0.265 km^2^ corresponds to the 1st quartile of home range sizes, while a home range size of 0.587 km^2^ corresponds to the 3rd quartile of home range sizes. Survival was higher for fish tagged in the fall than fish tagged in the spring.

**TABLE 2:** Regression coefficients, hazard ratios, standard error (SE) of the regression coefficients and *p*‐values from the Cox proportional hazards regression model

Parameter	Reg. coef.	Hazard ratio	SE	*p*‐value
Home range	−1.092	0.3355	0.3854	<0.01
Proportion of time in reserve	−28.77	3.19 × 10^−13^	9.775	<0.01
Season of tagging _Spring_	1.032	2.807	0.3754	<0.01
Home range:Proportion of time in reserve	2.29	9.87	0.7639	<0.01

Note

*N* = 69, number of events = 38.

## 4 DISCUSSION

Sea trout revealed individual consistency in home range size over a period of several months or even years, reflecting that home range can be considered an aspect of personality. Further, we found that home range size affected survival, and this relationship differed depending on the proportion of time the fish spent inside the reserve. For individuals that spent more than 48% of their time in the reserve, larger home ranges were associated with decreased survival, while individuals that spent less than 48% of their time in the reserve experienced increased survival with increasing home range size. In other words, the fitness landscape of sea trout appears to be influenced by spatial management, here represented by a no‐take marine reserve. As discussed below, this suggests that fish behaviour might evolve in response to fishing and therefore certain fishery management measures.

We found that home range size had a repeatability of 0.21, indicating that 21% of the variation in home range size is variation that occurs among individuals. This is comparable to the behavioural trait mean repeatability of 0.37 overall and 0.32 for fish previously reported in a meta‐analysis by Bell et al. (2009). Moreover, our results confirm previous studies showing repeatable home range in wild fish (0.43 for Atlantic cod *Gadus morhua*; Villegas‐Ríos, Réale et al., 2017; 0.33 for burbot *Lota lota*; Harrison et al., 2014), suggesting that consistent movement behaviour may be a general pattern for aquatic organisms. Individual variation in home range sizes has also been shown in mammals, for example for moose (*Alces alces*; van Beest et al., 2011). Repeatability was not detected for any depth‐related trait. Note that trout mainly utilized shallow depths, which means that we may not have been able to detect fine‐scale differences in depth use within the given accuracy and resolution of depth measurements. In future studies of fine‐scale depth use, tags with higher resolution would be advisable.

Our main finding is that the effect of home range size on survival differed depending on how much time the fish spent in the reserve. For sea trout that spent more than 48% of their time in the reserve survival decreased with increasing home range size. Here, a small home range implies spending little time in the fished area (i.e. at risk of being fished). Consistent removal of fish that strand out beyond reserve boundaries may eventually lead to selection against large home ranges in marine reserves (Villegas‐Ríos, Moland et al., 2017). For sea trout that spent less than 48% of their time in the reserve, survival increased with increasing home range. Note that this is the opposite pattern as previously found by both Alós et al. (2016) who reported selection against large home ranges in harvested areas, and Härkönen et al. (2014) finding that being explorative is linked to an increased vulnerability to angling in brown trout. Overall, there is contrasting evidence on the relationship between home range and surviving the fishery. Monk and Arlinghaus (2017) report no effect of swimming distance or activity space on vulnerability to capture by angling, while Olsen et al. (2012) report increased fishery survival for fish that displayed extensive horizontal shifts. Interestingly, individuals that express more risky behaviours in the wild, including exploration, experience increased survival (Moiron et al., 2020). Risky behaviours may lead to acquiring more resources followed by an increase in natural survival (Moiron et al., 2020). Having a larger home range size may be one such risky behaviour. Furthermore, it is less obvious why a large home range mainly located outside the reserve yields better survival than a large home range mainly located inside the reserve. A study with replicated protected‐unprotected area pairs could help to investigate whether the patterns found in this study are general patterns. These findings, combined with the fact that home range is repeatable and therefore likely partly heritable (Dochtermann et al., 2015), may entail evolutionary consequences for populations that are partially protected by marine reserves. That said, any local evolutionary change will also depend on the level of gene flow (Lenormand, 2002). In accordance with estimates of how much additive genetic variation contributes to personality, Dochtermann et al. (2015) estimated that the ratio of heritability to repeatability collected from literature averaged at 0.52 and ranged from 0 to 0.96 (Dochtermann et al., 2015). Few studies investigate heritability of behaviour in sea trout, but in a laboratory study on adfluvial brown trout, Kortet et al. (2014) found heritability of 0.14 (± 0.096) for the stress response ‘tendency to freeze’, but no heritability for boldness, exploration and aggression. To the best of our knowledge, there are no estimates of heritability of sea trout behaviour in the wild. Our paper is the first to present estimates of repeatability of sea trout behaviour in the wild, indicative of additive genetic variation (Dochtermann et al., 2015). In addition to additive genetic variation, repeatability may also reflect learning (Adriaenssens & Johnsson, 2011) and individual variation in utilizing heterogeneous environments (Bell et al., 2009). Hence, repeatability of home range size may also, to some degree, reflect individual differences in habitat use (Bell et al., 2009).

Body length affected all movement traits, with larger fish having larger home ranges, utilizing a larger range of depths and having a higher activity. As survival was affected by home range size, this may imply correlated selection on body length. However, body length did not affect survival directly. Home ranges were the largest in spring, and fish were more active during spring and summer than fall and winter. This is in accordance with sea trout intensifying their food search as temperatures increase during spring and summer (Klemetsen et al., 2003). Fish also swam deeper during spring and summer, which can be associated both with different habitat use and that the trout seek out colder water temperatures optimal for growth when surface temperatures rise (Eldøy et al., 2017; Kristensen et al., 2018). Fish tagged in the sea had larger home ranges, utilized a larger range of depths and had higher activity than fish tagged in the river.

Median survival in the wild was close to 11 months, and survival was higher for fish tagged in the fall. The latter could be explained by the upcoming spawning ascent, where sea trout will receive protection from fishing and experience a lower predation risk in the river (Thorstad et al., 2016). In general, trout survival is higher in freshwater as compared to sea (Solomon, 2006), and the duration of migration varies both within populations and among populations and latitudes (Klemetsen et al., 2003). This implies that yearly survival will vary substantially between river systems. Return rates from 193 sea trout tagged in the nearby river Storelva (<5 km from our study system) revealed 40% survival for trout spending one or two years at sea (Haraldstad, 2015). Survival might have been underestimated due to tag excretion, which would have led individuals to be falsely defined as dead. Also, there might be a negative effect of tagging on survival. A study on gastrically tagged salmonids found that small (9 mm) and large (13 mm) tags reduced survival from 94% in the control group to 90% and 72%, respectively (Kennedy et al., 2018). However, there are differences in tagging procedures between that study and our study, including tag positioning, time from capture to tagging and type of sedation agent, that may have affected survival.

Preserving a spectrum of different personalities may help sustain a population's resilience to environmental change, as different personalities are favoured across variable environmental conditions (Dingemanse et al., 2004). Interestingly, fish with different personalities adjust their behaviour differently when faced with environmental change (Villegas‐Ríos et al., 2018). Reactive fish (being less bold, exploratory and aggressive than proactive fish) may reduce their home ranges in response to increasing temperatures, while proactive maintain, or even slightly increase theirs (Villegas‐Ríos et al., 2018). This could further enhance potential fishing‐induced selection towards smaller home range sizes for proactive fish. Furthermore, spreading fishing effort over a range of habitats could help reduce capture bias, as different environmental conditions favour different behaviours (Killen et al., 2016). Lastly, a study of wild‐collected guppy populations showed that reproductive behaviour diversified in populations that were exposed to temporal heterogeneity in predator biomass (Barbosa et al., 2018). Reserves on the other hand provide spatial heterogeneity in ‘predator biomass’.

Our results have clear management implications. The fact that individually consistent home range size may affect survival differently inside and outside marine reserves implies that, ideally, a mosaic of marine reserves and areas (partially) open to harvest, representing a variation in fishing pressure, can provide a heterogeneous selection regime that can oppose directional selection on behaviour.

## CONFLICT OF INTEREST

None declared.

## Supporting information

Fig S1

Table S1‐S5

## Data Availability

Data are available from the Dryad Digital Repository: https://doi.org/10.5061/dryad.gqnk98sms
