# Supplementary material for: Selection on fish personality differs between a no‐take marine reserve and fished areas
Source: Evol Appl. 2021 May 1;14(7):1807–15. doi: 10.1111/eva.13242 (PMC8288012; doi:10.1111/eva.13242)
Supplement: Supplementary file 1 — Fig S1 [file EVA-14-1807-s001.docx]

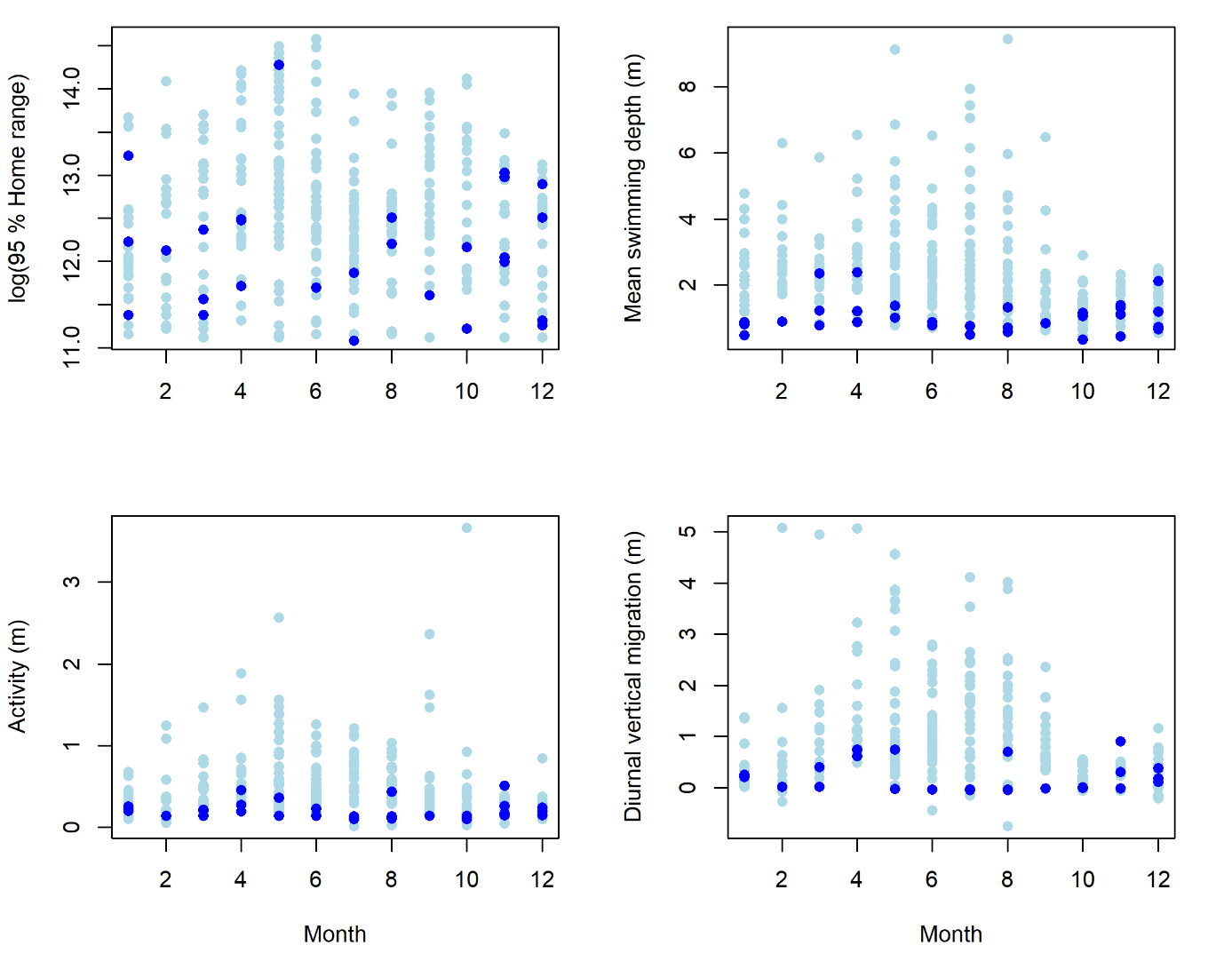


**Figure S.1** Seasonal development of the behavioural metrics home range, mean swimming depth, activity and diurnal vertical migration for fish tagged in the fjord (light blue) and in the river (dark blue) shown across months. Home range values are log-transformed monthly 95 % home ranges, mean swimming depth are monthly averages, activity values are standard deviation of depth per hour averaged over months and diurnal vertical migration is the daily difference between day and night averaged over months.
