# Supplementary material for: Selection on fish personality differs between a no‐take marine reserve and fished areas
Source: Evol Appl. 2021 May 1;14(7):1807–15. doi: 10.1111/eva.13242 (PMC8288012; doi:10.1111/eva.13242)
Supplement: Supplementary file 2 — Table S1‐S5 [file EVA-14-1807-s002.docx]

**Supporting information**

**Table S.1** Model selection for response variable home range size (log-transformed for normality). The random effects and correlation structure is selected in the first step and the fixed effects structure is selected in the second step. Model selection was done using AIC values. The best models from each selection step are shown in bold, and the selected model is enclosed.

| No | 1. Model structure | AIC |
| --- | --- | --- |
| **1** | **Home range = Season * Length * Capture location + Fish ID + COR_Month_** | **795.54** |
| 2 | Home range = Season * Length * Capture location + COR_Month\|Fish ID_ | 801.62 |
| 3 | Home range = Season * Length * Capture location + Fish ID | 810.23 |
| No | 1. Model structure | AIC |
| 1 | Home range = Season * Length * Capture location + Fish ID + COR_Month_ | 775.48 |
| **4** | **Home range = Season * Length + Capture location + Fish ID + COR_Month_** | **766.89** |
| 5 | Home range = Season * Capture location + Length + Fish ID + COR_Month_ | 771.34 |
| **6** | **Home range = Season + Length * Capture location + Fish ID + COR_Month_** | **767.72** |
| **7** | **Home range = Season + Length + Capture location + Fish ID + COR_Month_** | **767.07** |
| 8 | Home range = Season + Length + Fish ID + COR_Month_ | 772.43 |
| 9 | Home range = Season + Capture location + Fish ID + COR_Month_ | 769.15 |
| 10 | Home range = Length + Capture location + Fish ID + COR_Month_ | 783.78 |

**Table S.2** Model selection for response variable mean depth use. The random effects and correlation structure is selected in the first step and the fixed effects structure is selected in the second step. Model selection was done using AIC values. The best models from each selection step are shown in bold, and the selected model is enclosed.

| No | 1. Model structure | AIC |
| --- | --- | --- |
| **1** | **Mean depth use = Season * Length * Capture location + Fish ID**  **+ COR_Month_** | **1049.80** |
| **2** | **Mean depth use = Season * Length * Capture location + COR_Month\|Fish ID_** | **1050.10** |
| 3 | Mean depth use = Season * Length * Capture location | 1113.08 |
| No | 1. Model structure |  |
| 2 | Mean depth use = Season * Length * Capture location + COR_Month\|Fish ID_ | 1043.77 |
| **4** | **Mean depth use = Season * Length + Capture location + COR_Month\|Fish ID_** | **1032.67** |
| 5 | Mean depth use = Season * Capture location + Length + COR_Month\|Fish ID_ | 1041.66 |
| 6 | Mean depth use = Season + Length * Capture location + COR_Month\|Fish ID_ | 1039.60 |
| 7 | Mean depth use = Season + Length + Capture location + COR_Month\|Fish ID_ | 1037.91 |

**Table S.3** Model selection for response variable activity (log-transformed for normality). The random effects and correlation structure is selected in the first step and the fixed effects structure is selected in the second step. Model selection was done using AIC values. The best models from each selection step are shown in bold, and the selected model is enclosed.

| No | 1. Model structure | AIC |
| --- | --- | --- |
| **1** | **Activity = Season * Length * Capture location + Fish ID + COR_Month_** | **565.60** |
| **2** | **Activity = Season * Length * Capture location + COR_Month\|Fish ID_** | **563.60** |
| 3 | Activity = Season * Length * Capture location | 651.77 |
| No | 1. Model structure |  |
| 2 | Activity = Season * Length * Capture location + COR_Month\|Fish ID_ | 534.32 |
| 4 | Activity = Season * Length + Capture location + COR_Month\|Fish ID_ | 524.00 |
| 5 | Activity = Season * Capture location + Length + COR_Month\|Fish ID_ | 525.72 |
| **6** | **Activity = Season + Length * Capture location + COR_Month\|Fish ID_** | **522.79** |
| **7** | **Activity = Season + Length + Capture location + COR_Month\|Fish ID_** | **521.58** |
| 8 | Activity = Season + Length + COR_Month\|Fish ID_ | 525.96 |
| 9 | Activity = Season + Capture location + COR_Month\|Fish ID_ | 539.82 |
| 10 | Activity = Length + Capture location + COR_Month\|Fish ID_ | 539.56 |

**Table S.4** Model selection for response variable diurnal vertical migration. The random effects and correlation structure is selected in the first step and the fixed effects structure is selected in the second step. Model selection was done using AIC values. The best models from each selection step are shown in bold, and the selected model is enclosed.

| No | 1. Model structure | AIC |
| --- | --- | --- |
| **1** | **Diurnal vertical migration = Season * Length * Capture location + Fish ID + COR_Month_** | **680.83** |
| **2** | **Diurnal vertical migration = Season * Length * Capture location**  **+ COR_Month\|Fish ID_** | **678.83** |
| 3 | Diurnal vertical migration = Season * Length * Capture location | 730.25 |
| No | 1. Model structure |  |
| 2 | Diurnal vertical migration = Season * Length * Capture location  + COR_Month\|Fish ID_ | 662.17 |
| **4** | **Diurnal vertical migration = Season * Length + Capture location**  **+ COR_Month\|Fish ID_** | **650.85** |
| 5 | Diurnal vertical migration = Season * Capture location + Length  + COR_Month\|Fish ID_ | 659.87 |
| 6 | Diurnal vertical migration = Season + Length * Capture location  + COR_Month\|Fish ID_ | 658.81 |
| 7 | Diurnal vertical migration = Season + Length + Capture location  + COR_Month\|Fish ID_ | 656.86 |

**Table S.5** Model selection to predict survival of sea trout with a cox proportional hazards model. Model selection was based on AIC-values and done in two steps: (1) selecting the best model structure related to the main variables of interest (home range size, proportion of time spent in the reserve, tagging location), including interaction effects between home range size and proportion of time spent in the reserve or capture location, and (2) selecting the best model related to the additional covariates body length and season of capture. The best models from each selection step are shown in bold, and the selected model is enclosed.

| No | 1. Model structure | AIC |
| --- | --- | --- |
| 1  2 | Null model  Home range + Body length + Season of capture | 323.83  270.44 |
| 3 | Prop. of time in reserve + Body length + Season of capture | 270.08 |
| 4 | Capture location + Body length + Season of capture | 318.97 |
| **5** | **Home range * Prop. of time in reserve + Body length**  **+ Season of capture** | **265.82** |
| 6 | Home range + Prop. of time in reserve + Body length + Season of capture | 271.94 |
| 7 | Home range * Capture location + Body length + Season of capture | 269.90 |
| 8 | Home range + Capture location + Body length + Season of capture | 271.51 |
|  | 1. Model structure |  |
| **5** | **Home range * Capture location + Body length + Season of capture** | **265.82** |
| **9** | **Home range * Capture location + Season of capture** | **267.27** |
| 10 | Home range * Capture location + Body length | 273.90 |
| 11 | Home range * Capture location | 273.76 |
